# Silica based polishing of {100} and {111} single crystal diamond

**DOI:** 10.1088/1468-6996/15/3/035013

**Published:** 2014-06-24

**Authors:** Evan L H Thomas, Soumen Mandal, Emmanuel B Brousseau, Oliver A Williams

**Affiliations:** 1 Cardiff School of Physics and Astronomy, Cardiff University, Cardiff, UK; 2 Cardiff School of Engineering, Cardiff University, Cardiff, UK

**Keywords:** single crystal diamond, chemical mechanical polishing, diamond polishing, 81.05.U-, 81.05.ug

## Abstract

Diamond is one of the hardest and most difficult to polish materials. In this paper, the polishing of {111} and {100} single crystal diamond surfaces by standard chemical mechanical polishing, as used in the silicon industry, is demonstrated. A Logitech Tribo Chemical Mechanical Polishing system with Logitech SF1 Syton and a polyurethane/polyester polishing pad was used. A reduction in roughness from 0.92 to 0.23 nm root mean square and 0.31 to 0.09 nm rms for {100} and {111} samples respectively was observed.

## Introduction

Diamond has long been used for cutting and polishing applications due to its extreme hardness, high thermal conductivity and chemical inertness. However, properties such as a large band gap, high resistivity, high electron mobility, low dielectric constant, and low thermal coefficient of expansion make diamond an excellent candidate for high power–high frequency electronics [1, 2], and optical devices [3, 4]. With the advancement of technology it is now possible to economically synthesize high quality large area single crystal diamonds through homoepitaxial chemical vapor deposition (CVD) [5, 6]. However, to prevent defects and surface damage from the substrate from propagating into the CVD layer a polishing step is required [7, 8]. Recent experiments have also pointed to the presence of two dimensional hole gas on the surface of hydrogen terminated diamond [9]. To harness this phenomenon, and to fully utilize the properties of diamond in the applications mentioned above it is also important to have atomically flat, defect free top surfaces—necessitating an efficient polishing technique.

For the polishing of diamond, while many techniques exist including thermo-mechanical, ion beam, and thermal annealing, mechanical polishing has traditionally prevailed [10–15]. Mechanical polishing is typically done through the use of a fast rotating metal scaife charged with a diamond grit and olive oil binder. The sample is polished by applying it under pressures of 2.5–6.5 MPa for grinding and 1–2.5 MPa for polishing to a fast rotating cast iron scaife, resulting in linear velocities of approximately 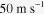 [16]. However, the polishing of diamond is highly anisotropic with two orders of magnitude difference in removal rate between hard and soft polishing directions for the {100} and {110} plane groups [17]. Along soft directions, polishing is the result of shearing between diamond chips on the scaife and the sample surface driving a phase conversion to non-sp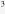 material [18]. As a result ‘nano-grooves’ are found on the surface with lengths of 20–1000 nm dependent on the grit used and depths of up to 20 nm, whereas, polishing along hard directions leads to fracturing along the {111} plane producing a rough ‘hill and valley’ type surface [19]. Polishing of the {111} plane meanwhile remains difficult [20], with only slight anisotropy between hard and soft directions [3]. Due to the inherent mechanical nature of this technique subsurface damage occurs, with fractures at the surface propagating into the bulk [21]. This problem of poor surface quality is often seen in the techniques mentioned above, preventing full use of the properties of diamond [22].

In order to reduce these polishing artifacts several methods have been proposed as a finishing technique, including chemo-mechanical [23–25] or tribochemical polishing [26], reactive ion etching [21], and UV photon based etching [8, 27]. In chemo-mechanical polishing a molten oxidizer is added, typically KNO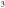 (potassium nitrate), NaNO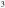 (sodium nitrate) [23], or H
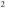
O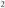 (hydrogen peroxide) [24]. Between the scaife and diamond sample hot spots of 360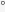 C are reached driving a conversion to CO and CO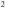 [23]. While this method achieves low roughness values, the use of scaife and elevated temperatures makes the process complicated and very different to that used in the silicon based electronics industry [28].

Previous work by the authors have shown chemical mechanical polishing (CMP), a technique used in the polishing of gate dielectrics in integrated circuit (IC) fabrication, can successfully be used to polish nanocrystalline diamond (NCD) films with a rate of 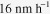 [29]. In this technique the sample is swept across a polyester/polyurethane based polishing pad doused with colloidal silica polishing fluid (Syton), without the use of any diamond grit or elevated temperatures. Drawing parallels with the mechanism used to describe the polishing of SiO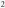 [30], it was tentatively suggested that wet oxidation of the diamond increases the hydroxide content on the surface, facilitating the binding of silica particles within the slurry. Should an asperity on the rough pad then create a sufficient shearing force on the silica particle, the particle and attached carbon atom will be removed, polishing the surface.

In this article the use of CMP on {100} and {111} single crystal diamond is demonstrated through the use of atomic force microscopy (AFM). The aspects of this adaption from the IC fabrication industry, including the condition of the polishing pad and post-polishing cleaning is also discussed.

## Experimental method

High pressure high temperature single crystal {100} and {111} samples were obtained from Element Six, with dimensions of approximately 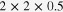 mm high and 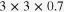 mm high respectively. Misalingment angles were stated to be less than 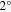. Before use both samples were given a standard SC-1 clean of 30% H
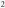
O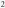 : NH
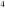
OH : deionized (DI) H
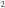
O (1 : 1 : 5) at 75 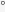
C for 10 min, followed by a ultrasonic DI H
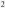
O bath for 10 min. In preparation for polishing, samples were bonded within a slight recess on a 2 inch polymer holder with cyanoacrylate. The recess was then filled up with Crystalbond 509 to prevent shear forces on crystal edges, ensuring a stable mounting while leaving only the surface to be polished protruding. This template was then placed inside a carrier chuck suitable for use with the CMP equipment.

CMP was carried out with a Logitech Tribo CMP tool equipped with a SUBA–X polyester/polyurethane pad and Syton colloidal silica alkaline polishing fluid (15–50% SiO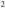, 9.2–10.1 pH, 4–5% ethylene glycol), as described elsewhere [29]. Before use an electroplated diamond grit conditioning plate was put into contact with the polishing pad to roughen the surface of the pad and maximize slurry distribution. During polishing both holder and pad were kept rotating at 60 rpm in opposite directions as the holder swept across the pad. Down pressure on the holder was maintained at 4 psi (27.6 kPa) while a backing pressure of 20 psi (138 kPa) was applied to prevent bowing of the holder and ensure contact between the diamond crystal and the polishing pad. The pad was also conditioned *in situ* with a second sweeping carrier chuck rotating at 60 rpm in the same direction as the sample, but at a reduced pressure of 1 psi (6.9 kPa). After initial wetting of the plate, the polishing slurry rate was maintained at 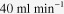. Polishing durations for the {100} and {111} single crystals were 3 and 7 h respectively, judged as the point at which sufficient removal of the nano-grooves was seen. After polishing, the samples were cleaned with SC-1 and hydrofluoric acid in an attempt to remove any organic contaminants and remaining silica.

AFM was performed with a Park Systems XE-100 AFM operating in non-contact mode equipped with NT-MDT NSG30 tips (320 kHz resonant frequency, 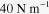 spring constant, 10 nm tip radius). Multiple areas of 25 *μ*m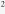 were scanned for each sample before and after polishing, with analysis of data being carried out by WSxM and Gwyddion SPM analysis software.

For analysis of the polishing pad, samples were taken of: a fresh pad, a conditioned pad, and a pad that had been subjected to 7 h of single crystal polishing. Scanning electron microscopy (SEM) images were taken with the secondary electron (SE2) detector of a Raith E-line SEM, operating at 10 kV accelerator voltage and 9 mm working distance.

## Results and discussions

### {100} single crystal

**Figure 1:**
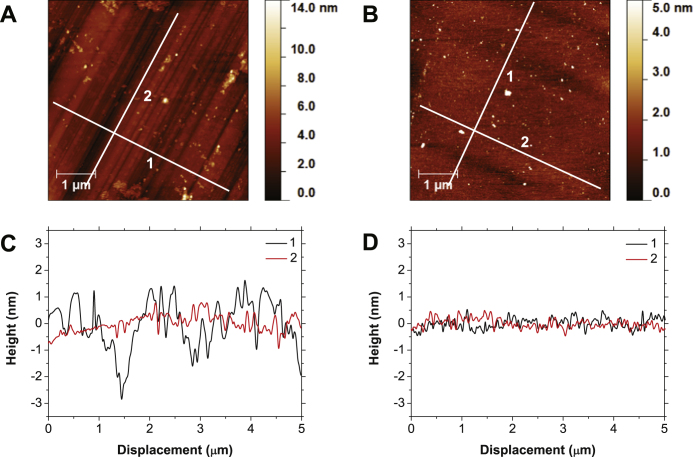
AFM images of a {100} orientated single crystal before (A) and after chemical mechanical polishing (B). Shown in panels (C) and (D) are line traces perpendicular to the mechanical polishing direction (1) and along the polishing direction (2) of the respective AFM images. Clear removal of the nano-grooves can be seen in the AFM images, backed up by the dramatic reduction in amplitude of the perpendicular to polishing direction line trace.

Typical AFM scans of the {100} single crystal before and after polishing are shown in figures 1(A) and (B), while lines traces perpendicular to the original polishing direction (1), and along the polishing direction (2) are plotted in figures 1(C) and (D). As can be seen in figures 1(A) and (C), the surface of the sample prior to CMP consists of clearly defined nano-grooves as a result of phase transformation along the 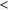 100 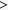 soft direction. From the 5 *μ*m perpendicular line trace it can be seen that the nano-grooves widths are between 100 and 500 nm, with an average depth of 3 nm. Meanwhile, for the trace along the polishing direction less variation is seen with little indication of fracture. Roughness values are 0.92 nm root mean square (rms) and 0.34 nm rms for the perpendicular to and along polishing direction line traces respectively.

Looking at the 3 h CMP polished AFM image and line traces of figures 1(B) and (D) a clear difference can be seen with the removal of the grooved features evident in 1(A) and (C) to the point at which it is difficult to resolve the original polishing direction. The line trace perpendicular to the polishing direction shows a decrease in the larger undulations of the nano-grooves to a point at which it is in close agreement with the trace taken along the original polishing direction, with roughness values being 0.23 and 0.19 nm rms respectively. It is also worth noting that a lack of polishing debris can be seen in the polished AFM image, showing the ease of removal of the polishing slurry used.

### {111} single crystal

**Figure 2:**
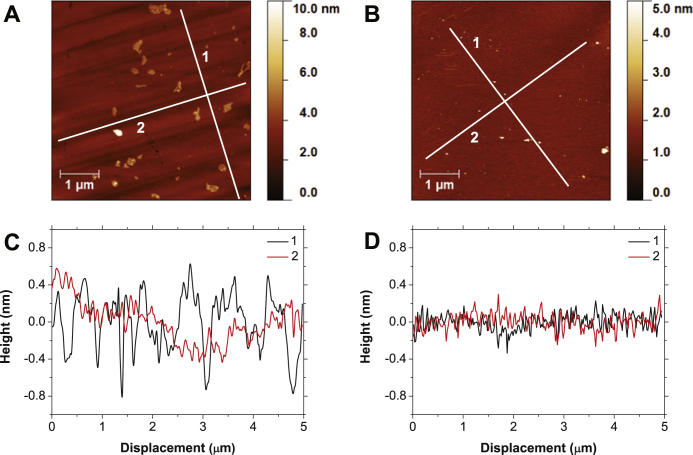
AFM images of the {111} polished plane before (A) and after chemical mechanical polishing (B). Shown in panels (C) and (D) are line traces similar in fashion to those seen in figure 1. Once again, a clear removal of grooved surface features can be seen, leading to a very smooth surface with line trace roughness reducing from 0.31 and 0.23 nm rms for perpendicular to and along original polishing direction to 0.09 nm rms.

In a similar fashion to the {100} single crystal sample, figure 2 shows typical AFM images of the {111} single crystal before and after being subjected to 7 h of CMP along with line traces perpendicular (1), and along the mechanical polishing direction (2). As can be seen in figure 2(A), the {111} single crystal has similar, but shallower, grooved features as the {100} crystal. Roughness values seen perpendicular to and along the original polishing direction are 0.31 nm and 0.23 nm rms respectively. After being subjected to CMP it is again very difficult to determine the original polishing direction due to the dramatic reduction in the grooved features. Looking at the two perpendicular line traces they appear very similar, reiterating the removal of these grooves. Roughness values for these traces are significantly lower at 0.09 nm rms for both perpendicular and along the polishing direction traces.

### Polishing pad

As stated earlier, the tentative model used to describe the CMP of diamond, and the model to describe traditional CMP on which it is based, rely on shearing forces being applied to bound silica particles. Therefore it would be expected that the condition and properties of the polishing pad heavily dictate the wear rate [31–34]. In traditional CMP it has been seen that the removal rate is directly related to the surface roughness and hence the number of surface asperities [34]. However, the pad also needs to be porous to allow for slurry distribution and clearing away of spent material. Therefore, before use pads are typically ‘run in’ [33] or ‘conditioned’. In this process an electroplated diamond grit plate is swept across the rotating polishing pad abrading the soft polymer material resulting in an increase in surface roughness and opening up of pores. Upon use in polishing the pad is then plastically deformed by the sample, closing pores and reducing the number of these surface asperities in as little as 10 min [33]. This decrease limits the shearing forces applied to the bound silica particles, leading to a reduction in the polishing rate. While the diamond samples themselves will condition the pad surface, due to the polishing duration being in the hours rather than minutes [33], *in situ* conditioning of the pad is also needed and has been carried out in the process described within this article. With the difference in mechanical properties of diamond and the materials traditionally polished with these polyester/polyurethane pads, work is needed to characterize the pad during diamond polishing.

**Figure 3:**
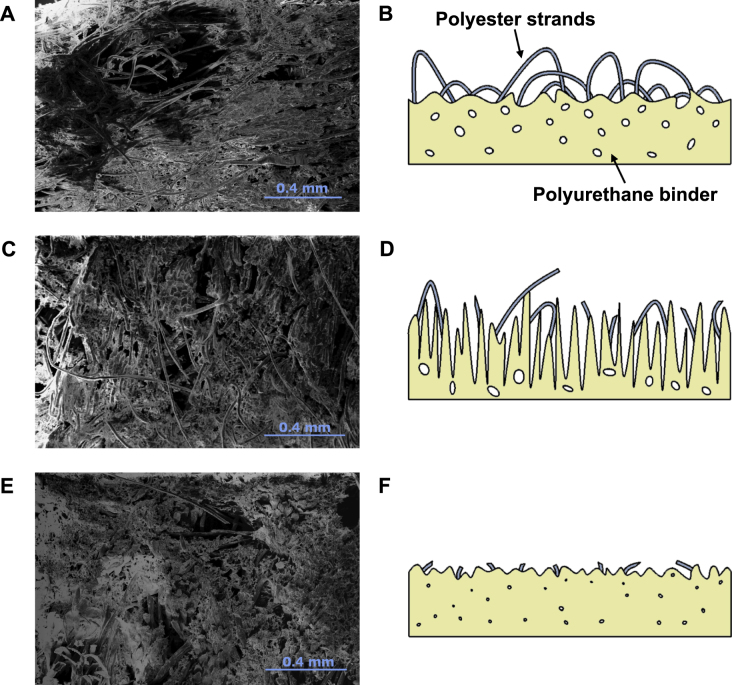
Plan view SEM images of polishing pad samples taken: (A) before use, (C) after conditioning for 1 h, and (E) after 7 h of single crystal polishing. Shown in panels (B), (D), and (F) are corresponding side view schematics of the polishing pad at the before use, conditioned, and used stages respectively. Abrasion of the pad by the electroplated diamond grit conditioner can be seen after 1 h of conditioning, with a general increase in the surface roughness of the polyurethane foam binder. Also visible is the severing of the polyester strands. After 7 h of use the pad has become severely abraded leading to a reduction in the polishing rate along with nearly all polyester strands being cut.

Plan view SEM images of an as received SUBA-X polishing pad, a conditioned pad, and a pad subjected to 7 h of single crystal CMP can be seen in figures 3(A), (C), and (E). Also shown in panels (B), (D), and (F) are corresponding side view schematics of the polishing pad at the different stages of use. From the fresh pad and the corresponding schematic, highly orientated polyester strands can be seen to be bound together by dense polyurethane foam. After being conditioned, the abrasion by the electroplated diamond grit can be seen; the polyester strands show signs of being severed by the conditioner while the polyurethane foam has also opened up and become rougher. It can therefore be assumed that conditioning has increased the surface asperities on a scale closer to the size of the silica particles within the slurry. After 7 h the SEM image shows significant wear with most of the polyester strands being severed and the polyurethane foam appearing severely abraded, signifying that at this point conditioning is no longer affective. Pores within the material have also closed up, preventing efficient slurry distribution and removal of spent material. Wear tracks were also visible by eye after 7 h, reiterating this considerable wear.

### Discussion

Looking at the AFM images for the {100} and {111} orientated single crystals of figures 1 and 2, a clear polishing effect can be seen. The deep nano-grooves left over from mechanical polishing have been removed, leaving a smoother surface over the 25 *μ*m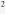 image area. The line trace perpendicular to these polishing grooves reiterate this with a reduction in roughness from 0.92 to 0.23 nm rms and 0.31 to 0.09 nm rms for the {100} and {111} samples respectively. After polishing the line traces appear very similar to the traces along original polishing direction, again showing this clear removal. With regards to polishing rate, it can tentatively be said these roughness values show faster polishing on the {100} than the {111} plane, as also seen in mechanical polishing. To describe this polishing on NCD a model of wet oxidation of the surface by the slurry, followed by binding of silica particles and subsequent shearing away has been proposed elsewhere [29]. Given the results presented within this paper, it is believed the same model can be used to describe the CMP of single crystal diamond.

It is worth noting that no attempt was made at polishing along soft mechanical directions, with the sample being rotated while sweeping across the pad. Equal time was then spent polishing along hard and soft polishing directions with no indication of the fracture damage seen when mechanically polishing along hard directions [3], showing the gentle nature of the polishing mechanism used here. The surfaces are also free of polishing debris or remaining polishing slurry due to the combined use of SC-1 and HF cleaning after polishing, while it can also be assumed that there has been little to no increase in subsurface damage due to the lack of diamond grit being used in the polishing slurry. Due the difference in properties of diamond and the materials typically polished with CMP, study of the polishing pad is needed to optimize the adapted technique. Due to the hardness of diamond additional abrasion of the pad is seen, as highlighted in figure 3(E), reducing the life time of the pad.

It has been shown that CMP can be used efficiently to remove the nano-grooved artifacts brought about through mechanical polishing of single crystal diamond and provide low roughness {100} and {111} diamond surfaces. With this technique polyester/polyurethane pads are used at room temperature with low applied pressures, without the use of diamond grit, simplifying the process and making CMP a promising technique.

## Conclusion

{100} and {111} orientated single crystal diamond has been polished with CMP through the use of a polyester/polyurethane pad and an alkaline colloidal silica polishing fluid. Clear removal of the mechanical polishing induced nano-grooves can be seen, with {100} surface roughness values being reduced from 0.92 to 0.23 nm rms along a 5 *μ*m line trace taken perpendicular to the direction of the nano-grooves. Meanwhile, the equivalent line trace on the {111} sample shows a reduction from 0.31 to 0.09 nm rms. Therefore with its simplicity due to the use of materials commonly found in the IC fabrication industry, along with the lack of diamond grit and elevated temperatures, CMP is a promising technique for removing mechanical polishing introduced artefacts.
